# Understanding the role of psychological factors in long COVID: a network analysis approach

**DOI:** 10.1093/eurpub/ckag038

**Published:** 2026-03-20

**Authors:** Sofia-Marie Oehlke, Andreas Goreis, Annika Lozar, Diana Klinger, Paul L Plener, Michael Zeiler, Oswald D Kothgassner

**Affiliations:** Department of Child and Adolescent Psychiatry, Medical University of Vienna, Vienna, Austria; Department of Child and Adolescent Psychiatry, Medical University of Vienna, Vienna, Austria; Department of Child and Adolescent Psychiatry, Medical University of Vienna, Vienna, Austria; Department of Child and Adolescent Psychiatry, Medical University of Vienna, Vienna, Austria; Department of Child and Adolescent Psychiatry, Medical University of Vienna, Vienna, Austria; Department of Child and Adolescent Psychiatry and Psychotherapy, University of Ulm, Ulm, Germany; Department of Child and Adolescent Psychiatry, Medical University of Vienna, Vienna, Austria; Department of Child and Adolescent Psychiatry, Medical University of Vienna, Vienna, Austria

## Abstract

Long COVID (LC) is a heterogeneous, multisystem condition that persists beyond the acute phase of SARS-CoV-2 infection. Psychological symptoms are highly prevalent and may influence the course and severity of LC. However, their specific role within the broader symptom structure remains insufficiently understood. This study applied a psychological network approach to examine how psychological factors contribute to the overall symptom structure of LC and to identify central and bridging variables that may serve as promising targets for intervention. A sample of 283 individuals with LC (*n*_female_ = 235, *n*_male_ = 47, *n*_diverse_ = 1; age: *M *= 39.48, *SD *= 13.29) completed an online survey assessing post-viral physical symptoms and psychological factors, including depression, anxiety, COVID-19-related traumatic stress, and lack of self-efficacy. A regularized partial correlation network was estimated based on ten variables. The network revealed a dense degree of connectivity, with psychological factors integrated into the broader symptom structure. Depression emerged as the most central variable. Cardiovascular and respiratory symptoms, neurological symptoms, and depression served as key bridge variables. Lack of self-efficacy showed moderate associations with COVID-19-related traumatic stress and anxiety. Female gender was linked to greater gastrointestinal symptom burden, while older age was associated with more pronounced cardiovascular and respiratory symptoms. This study underscores the central role of psychological factors—particularly depression—as key targets for intervention in LC. By advancing the understanding of factors shaping health outcomes in LC, our findings support the integration of psychological approaches into the clinical management of affected individuals.

Key points

- Psychological symptoms are common in long COVID, but their role within the broader symptom structure has been unclear; this study addresses this gap by using a psychological network approach to examine how psychological and physical symptoms are interrelated.
- The network analysis revealed that psychological factors and physical symptoms do not occur in isolation but are closely connected within an integrated system, suggesting that long COVID involves systemic symptom interactions.
- Depression emerged as the most central symptom, highlighting its potential role in maintaining overall symptom burden and its relevance as a clinical intervention target.
- Depression, together with cardiovascular, respiratory, and neurological symptoms, linked psychological and physical symptom clusters, pointing to possible overlaps in their underlying processes.
- These findings underline the importance of addressing psychological factors—particularly depression—in treatment planning for long COVID and suggest the need for longitudinal and interventional research to evaluate the effectiveness of mechanism-based interventions.

## Introduction

Long COVID (LC) has emerged as a complex and persistent condition following SARS-CoV-2 infection [1]. According to the National Institute for Health and Care Excellence, LC refers to the continuation or emergence of symptoms beyond four weeks after acute infection, encompassing both ongoing symptomatic COVID-19 (4–12 weeks) and post-COVID-19 syndrome (>12 weeks) [2]. LC presents with a highly heterogeneous, multisystemic symptom profile [1]. Meta-analytic data identified fatigue, dyspnoea, arthromyalgia, sleep disturbances, cognitive impairment, depression, and anxiety as the most common symptoms [3].

Despite increasing research efforts, the mechanisms underlying LC remain poorly understood. Most models emphasize physiological consequences of SARS-CoV-2 infection but tend to neglect the role of psychological factors in symptom dynamics and persistence [4]. Nevertheless, depression and anxiety have been linked with an increased risk of developing LC, pointing to the potential involvement of psychological vulnerability in symptom progression [4]. Moreover, in individuals with LC, mental health disturbances such as anxiety, depression, and post-traumatic stress not only frequently co-occur with physical complaints, but are also associated with greater overall symptom severity and functional impairment [4]. Understanding the role of psychological factors within the broader symptom architecture of LC is crucial for refining how the condition is approached and treated. Moreover, given the modifiability of psychological factors, their integration into diagnostic processes and treatment planning holds significant potential for improving health outcomes in LC.

Psychological network analysis is a methodological framework for modelling complex symptom structures [5]. Unlike traditional variable-centred approaches, psychological network models emphasize the interrelations among symptoms—how they co-occur, mutually reinforce, and sustain the condition—thereby indicating leverage points for targeted treatment [5]. Only a few studies have applied network analysis to LC. Most have focused on narrow symptoms domains such as neurocognitive symptoms [6], sensory disturbances [7], or overall symptom count [8]. Others have excluded psychological factors altogether [9, 10]. Findings have varied considerably, likely due to differences in symptom selection, sample composition, and analytic strategies. Nonetheless, affective symptoms—particularly depression—consistently emerge as central within LC networks, suggesting a pivotal role in maintaining and amplifying the condition [6, 8, 11]. To date, only one preprint study has modelled both psychological factors and a broad array of physical symptoms [11]. That analysis reported dense symptom interconnectivity, with affective and sensory symptoms displaying high centrality.

Building on this work, the present study applied psychological network analysis in individuals with LC to model a broad range of guideline-based physical symptom alongside key psychological factors, including depression, anxiety, post-traumatic stress related to the COVID-19 pandemic, and lack of self-efficacy. Our primary objective was to specify how psychological factors are integrated within the overall symptom network. Additionally, we aimed to identify central nodes within the network as well as nodes bridging physical and psychological symptoms, expecting affective symptoms to play a prominent role based on previous research [6, 8, 11]. This approach seeks to advance understanding of the heterogenous symptom structure of LC and how it is potentially shaped by psychological factors, thereby informing more targeted, mechanism-based interventions.

## Methods

### Procedure and participants

All references for the methods used in the present study are provided in the Supplementary Material. The present study is a secondary analysis based on data from a previously conducted cross-sectional survey investigating mental health outcomes among three groups: (i) individuals with LC, (ii) individuals with a history of SARS-CoV-2 infection without post-viral symptoms, and (iii) individuals with no prior SARS-CoV-2 infection [S1]. For the current analysis, only participants meeting the criteria for LC were included.

Participants were recruited online through various social media platforms. Eligibility criteria comprised: (i) a minimum age of 14 years, and (ii) fluency in German. Informed consent was obtained electronically in accordance with the European General Data Protection Regulation (GDPR). The survey, which took approximately 10 minutes to complete, offered no financial or material incentives. Data collection took place between June 2021 and January 2022. Of the 635 individuals who provided informed consent, 81 were excluded due to incomplete responses on key measures, and five due to implausible or inconsistent response patterns. The overall sample consisted of *N *= 549 participants. LC was classified according to the NICE criteria, defined as at least one symptom persisting for more than four weeks after acute SARS-CoV-2 infection and not explained by another medical condition [2]. Based on this definition, *N *= 283 participants met the LC criteria and were included in the present analysis. Table 1 displays the demographic and COVID-19 specific clinical characteristics of the respective participants. The study was conducted in accordance with the Declaration of Helsinki and received ethical approval from the Ethics Commission of the Medical University of Vienna (Reference number: EK 1433/2021). During the preparation of this work the authors used ChatGPT to improve the clarity and fluency of the manuscript. No content was generated or interpreted by the tool. After using this service, the authors reviewed and edited the content as needed and take full responsibility for the content of the publication.

**Table 1.** Demographic and COVID-19-specific sample (*N *= 283) characteristics

Variables	% (*n*) *M* (SD)
Age, range	39.48 (13.29), 14-69
Age groups	
<30 years	27.2% (77)
31–60 years	69.2% (196)
>60 years	3.5% (10)
Gender identity	
Female	83% (235)
Male	16.6% (47)
Other	0.4% (1)
Economic status^a^	
<500€	9.3% (25)
501–1100€	17.8% (48)
1101–1500€	12.3% (33)
1501–3000€	37.9% (102)
>3000€	17.8% (48)
No regular income	4.8% (13)
Citizenship	
Austria	64.6% (106)
Germany	29.9% (49)
Switzerland	1.2% (2)
Other	2.4% (4)
Employment status prior to COVID-19 pandemic	
Student	15.2% (43)
Educational leave	1.1% (3)
Maternity leave	0.7% (2)
Leave due to childcare	1.8% (5)
Self-employed	9.2% (26)
Salaried employed	65.4% (185)
Unemployed	2.1% (6)
Other	9.5% (27)
COVID-19 vaccination	
None	36.1% (99)
One	26.3% (72)
Two	37.6% (103)
Acute COVID-19 severity	
Asymptomatic	4.4% (12)
Mild	47.1% (129)
Moderate	39.8% (109)
Severe	6.6% (18)
Hospitalization	2.2% (6)
Symptom-free period following acute COVID-19	
No symptom-free period	44.9% (127)
1 week	18.7% (53)
4 weeks	19.4% (55)
8 weeks	9.9% (28)
Duration of long COVID (in weeks)^b^	33.43 (33.18)
Impaired daily functioning due to long COVID	
Strong	62.5% (177)
Moderate	28.3% (80)
None	7.8% (22)

a Economic status represents the average monthly disposable income.

b Duration of post-viral symptoms in weeks since their onset following acute COVID-19.

### Materials

#### Node selection for the network analysis

The variables included in the psychological network analysis (subsequently referred to as *nodes*) comprised scores derived from standardized self-report questionnaires (for detailed information see Supplementary Material) as well as gender (female vs. non-female) and age, as female gender and advanced age represent established risk factors for LC [S2].

##### Psychological factors

Psychological distress during the past week was measured using the German-validated short version of the Brief Symptom Inventory (BSI-18; [S3]). COVID-19 related traumatic stress was assessed over the past week using the German adaptation of the Impact of Event Scale for COVID-19 (IES-COVID-19; based on [S4]; validated by [S5]). The IES is a well-established self-report scale assessing subjective traumatic stress related to a specific life event [S5]. The IES-COVID-19 adapts this instrument to specifically reference the COVID-19 pandemic as the stressor, capturing the severity of pandemic-related traumatic stress. Last, chronic stress over the past month–including two subscales, helplessness and self-efficacy–was assessed using the validated German version of the Perceived Stress Scale (PSS; [S6]). Self-efficacy items were reverse-coded (subsequently referred to as *lack of self-efficacy*).

##### Post-viral symptoms

Post-viral symptoms were assessed using 21 items derived from an adapted German version of the Coronavirus Infection Survey Questionnaire (CIS; [S7]). Figure S1 in the Supplementary Material displays the proportions of individual post-viral symptoms reported. Post-viral symptoms were then grouped into domains based on an exploratory factor analysis (for more details and results, **see Supplementary Material, Table S1**). The analysis revealed five symptom domains: (i) neurological symptoms, (ii) cardiological and respiratory symptoms, (iii) gastrointestinal symptoms, (iv) sensory symptoms, and (v) psychological symptoms. Although the fifth domain—psychological symptoms—emerged as a distinct factor, it was excluded from the network model to avoid conceptual overlap with the broader psychological constructs already captured through other self-report questionnaires (e.g. BSI-18). For the four remaining physical symptom domains, we calculated a composite score per participant by summing the number of reported symptoms within each domain.

### Data analyses

We used *IBM SPSS* version 29 for Windows (IBM Corporation, Armonk, NY, USA) to summarize sociodemographic and COVID-19 specific characteristics as well as conducting an exploratory factor analysis. Further statistical analyses were performed using *R* (version 4.4.0).

#### Missing data

First, we explored the amount of missing data for variables considered to be included in the network analysis. The percentage of missing data across all variables and individuals was low (2.8%) with answers regarding COVID-19 related post-traumatic stress showing the highest percentage of missing data (17%). Little’s MCAR test suggested that the missing data were consistent with a missing-completely-at-random assumption [*χ*^2^(107) = 132.0, *P* = .051]. Thus, missing data were handled using multiple imputation (predictive mean matching, 5 imputations, 50 iterations) using the *mice* package (version 3.16.0) [S8].

#### Topological overlap between selected nodes

Next, we used the *goldbricker* function implemented in the *R* package *networktools* (version 1.5.2) to explore potential topographical overlap between the variables considered for the network analysis and thus, to identify redundant nodes [S9]. This function computes the proportions of correlations that significantly differ for each pair of nodes (node pairs that differ in < 25% of their correlations to other nodes were considered as redundant, a *P* value of .05 was used to determine statistical significance). This analysis revealed a topographical overlap of the *helplessness* subscale of the PSS with other nodes in the network (*depression* and *anxiety* assessed by the BSI-18) and thus, this variable was excluded from the network model. Bivariate correlation coefficients between these variables are provided in Fig. S2 in the Supplementary Material.

#### Network estimation

We estimated and visualized regularised partial correlation networks using the *R* packages *qgraph* (version 1.9.8) and *bootnet* (version 1.6) by applying the graphical least absolute shrinking and selection operator (gLASSO) in combination with the Extended Bayesian Information Criterion (EBIC) [S10-S13]. By using this approach, small or unstable correlations between nodes are set to zero which produces a more parsimonious and better interpretable network. In the network plot, nodes that are closely interconnected are clustered, nodes with a high number of associations to other nodes are placed in the centre of the plot while nodes with rather weak associations to other nodes are placed in the periphery. The strength of partial correlations between the nodes (called *edges*) are represented by the line thickness while positive associations are shown in green and negative associations in red.

#### Measures of node centrality and bridge nodes

As a measure of node centrality, we calculated *one-step expected influence* (z-standardized) which quantifies the weighted number and strength of all connections of a specific node while taking positive and negative associations into account [S14]. Of note, as we observed almost only positive associations, this measure of node centrality is equivalent to *strength* which is also often reported in network analyses. Furthermore, we used the *bridge* function of the *networktools* package to identify bridge symptoms between predefined clusters (here called *communities*) of variables which were the nodes on physical symptom domains, psychological factors and sociodemographic variables. We calculated the one-step bridge expected influence which quantifies the summed edge weights of a node of interest to all other nodes that are not in the same community.

#### Network stability and edge accuracy

Finally, a case-dropping subset bootstrap approach (*n *= 1.000 boots) implemented in the package *bootnet* was applied to estimate the stability of the central index of one-step expected influence. We further calculated the correlation stability (CS) coefficient which should be above 0.5 [S10]. Non-parametric bootstrapping (1.000 boots) was performed to evaluate edge weights accuracy by calculating 95% confidence intervals.

## Results

### Network estimation

**Figure 1.**
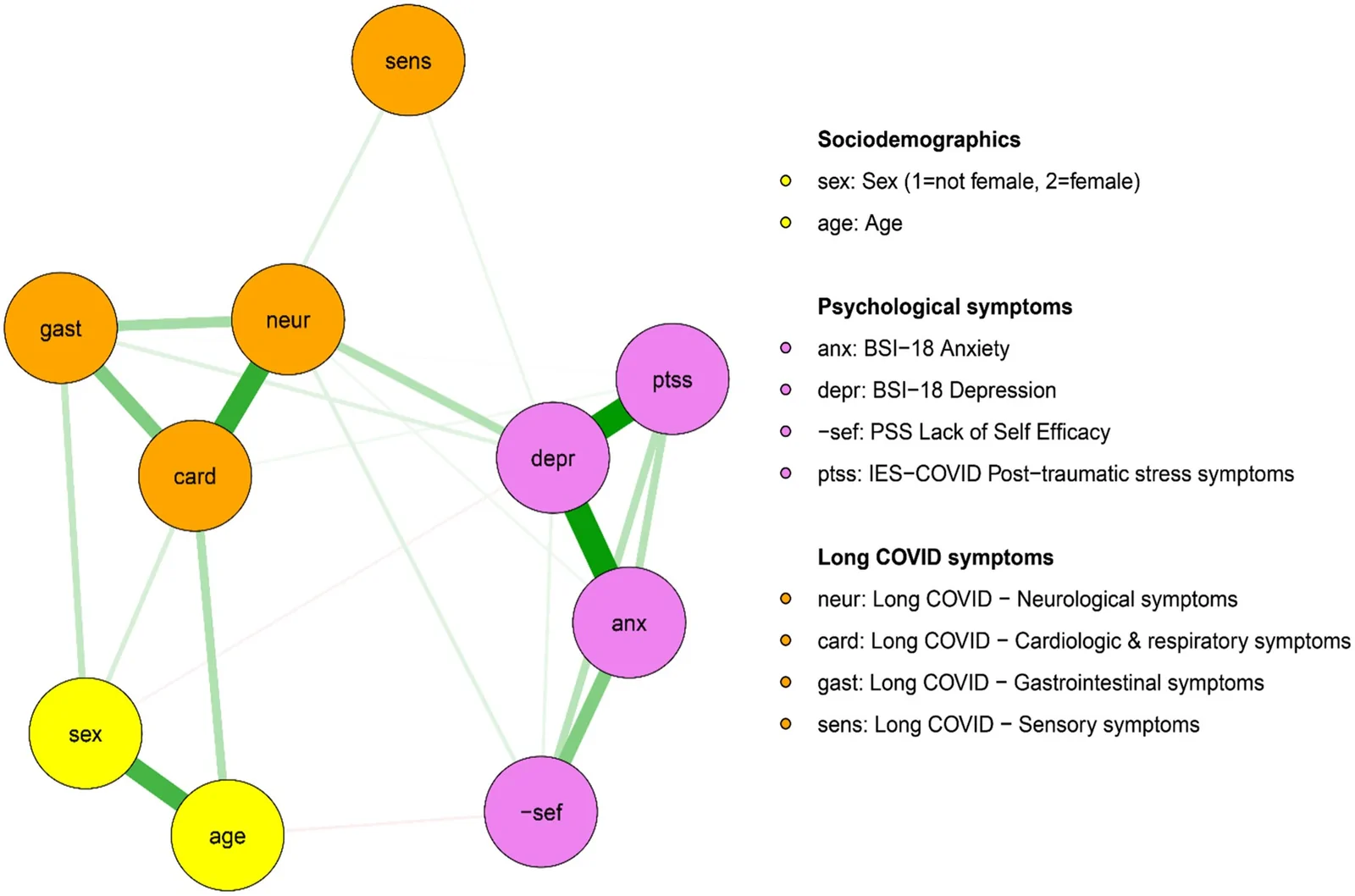
Network structure based on graphical least absolute shrinking and selection operator (gLASSO) regularised partial network estimation.

The results of the gLASSO regularised partial network are shown in Fig. 1. The network plot displays a cluster of nodes representing physical symptom domains (cardiologic and respiratory, neurologic and gastrointestinal symptoms) as well as a cluster of psychological factors (depression, anxiety, COVID-19-related traumatic stress, lack of self-efficacy) while nodes within these clusters were closely interconnected. Sensory symptoms appeared more peripheral, showing only weak associations with neurological symptoms and depression. Higher levels of depression were linked to greater neurological symptom severity. Lack of self-efficacy showed moderate associations with anxiety and COVID-19-related traumatic stress. Female gender was positively associated with gastrointestinal symptoms, while higher age was related to more pronounced cardiologic and respiratory symptoms.

### Node centrality and bridge expected influence

**Figure 2.**
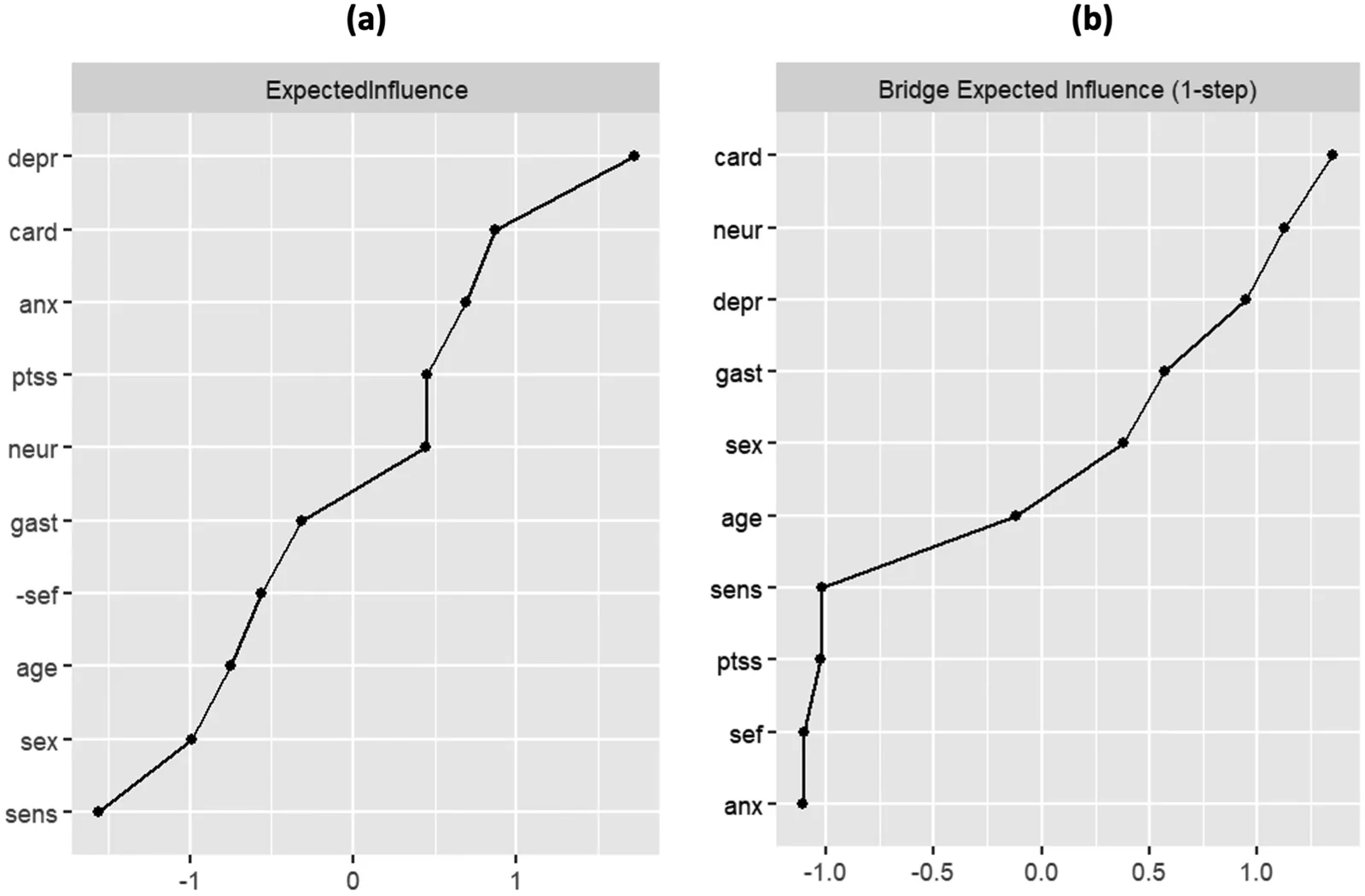
Standardized node centrality (z-scores) including (a) one-step expected influence and (b) bridge expected influence. For an explanation of node abbreviations, see legend of Fig. 1.

As shown in Fig. 2a, depression was the node with highest expected influence in the network followed by cardiologic/respiratory symptoms and anxiety. Three nodes exhibited high bridge expected influence (cardiologic/respiratory symptoms followed by neurological symptoms and depression) indicating that these nodes act as a bridge between the communities (see Fig. 2b). The standardized centrality indices are provided Table S2 in the Supplementary Material.

### Network stability and edge accuracy

Case-dropping subset bootstrapping revealed high stability of the central index measure of one-step expected influence (see Fig. S3 in the Supplementary Material). The CS coefficient was 0.734 indicating that the network has high reliability and reproducibility. Figure S4 in the Supplementary Material shows 95% confidence intervals for edge weights derived from non-parametric bootstrapping. Confidence intervals of edge weights were not excessively large; thus, they can be regarded as sufficiently accurate to be interpreted.

## Discussion

This study applied a psychological network analysis to explore the role of psychological factors in LC and to examine their interrelations with physical symptom domains. Both physical and psychological symptom clusters appeared cohesive and were well-integrated into the overall network, despite their predefined structure. In line with our expectation, depression emerged as the most central node, showing its strongest association with neurological symptoms among all physical symptom domains. Cardiovascular and respiratory symptoms served as strongest bridging node, followed by neurological symptoms and depression, identifying them as key nodes linking the physical and psychological symptom clusters.

The marked interconnections observed in this study contribute to the ongoing debate about whether LC represents a single, coherent syndrome or an overarching term for conditions with distinct clinical manifestations that partially overlap [11]. While previous research has employed clustering techniques to delineate separate symptom domains [12], the present network analysis examined their joint organization and revealed distinct connectivity among them. Rather than functioning in isolation, physical symptom domains appear to interact as part of an integrated symptom system. From a network perspective, this underscores the relevance of treatment strategies that focus on symptoms with high centrality or bridging potential, as these may exert broader influence across the overall network [5].

The central role of psychological factors in the present network aligns with growing evidence that psychological and physical symptoms in LC are dynamically interrelated [4]. Previous network analyses report similar patterns, with psychological symptoms embedded within the broader structure [6, 11, 13]. Depression emerged as the most central node in our analysis, consistent with prior findings [6, 8, 11]. In network models, central symptoms are assumed to exert disproportionate influence; once activated, they are more likely to trigger other symptoms and contribute to escalating symptom dynamics [5]. Depression may therefore act as a hub that shapes how symptoms cluster, persist, and spread across the clinical presentation of LC. Beyond its structural relevance, depression is highly prevalent in LC—affecting up to 13% of individuals across post-acute stages [3]—and is associated with greater symptom-related impairment [14]. Targeting depression may thus offer both preventive and therapeutic advantages. Recent trials of cognitive behavioral therapy (CBT) in LC, though not primarily focusing on depression, have shown moderate improvements in physical functioning, fatigue, and disease coping [15, 16]. Given CBT’s established efficacy for depression in other populations [17], its application in LC may benefit from a stronger focus on depressive symptoms.

Notably, depression displayed its strongest association with neurological symptoms. While depression may partly reflect a psychological reaction to chronic illness, emerging evidence suggests it also interplays with underlying neurobiological mechanisms [18]. Pathophysiological processes such as neuroinflammation and blood-brain barrier disruption, which are implicated in the pathology of LC [19], are also established drivers of depressive symptomatology [20, 21]. From this perspective, targeting neurological alterations in LC may also alleviate depressive symptoms, which, given depression’s central role in shaping the overall clinical picture, could in turn lead to broader improvements in patient outcomes. To support this notion, studies evaluating neurological interventions in LC should systematically assess depressive outcomes alongside primary neurological endpoints.

Cardiovascular and respiratory symptoms, neurological symptoms, and depression emerged as key bridging nodes linking physical symptom domains and psychological factors. In network theory, bridging nodes connect otherwise distinct clusters and enable interactions that contribute to sustaining the broader symptom network [5]. Notably, the identified bridging symptoms have each been linked to autonomic dysregulation, an established pathophysiological mechanism in LC [1]. The autonomic nervous system regulates involuntary physiological processes by dynamically balancing sympathetic and parasympathetic activity in response to internal and external demands [22]. When this regulatory capacity is dysregulated, adaptive responses may become excessive or blunted, potentially giving rise to persistent post-viral symptoms [23]. Although autonomic dysfunction is widely considered a hallmark of LC, empirical findings regarding its specific patterns remain heterogeneous. While some studies report indices of sympathetic predominance [24, 25], others have observed increased parasympathetic activity [26], underscoring the complexity of autonomic alterations in this condition.

At a symptom level, autonomic dysregulation has been implicated in cardiovascular, respiratory, and neurological manifestations in LC, including fatigue, orthostatic intolerance, palpitations, and dyspnoea [27]. In addition, autonomic regulation plays a central role in interoceptive processing and mood regulation, and its disruption is frequently observed in depression and other psychiatric conditions [30]. Autonomic dysregulation may therefore represent a transdiagnostic mechanism through which physical and psychological symptoms in LC interact and mutually reinforce one another. As a central interface of psychophysiological regulation, the autonomic nervous system enables continuous bidirectional crosstalk between psychological states and peripheral physiological processes [28]. Via this pathway, psychological symptoms may exacerbate physical symptom burden by precipitating or amplifying autonomic instability [22]. Conversely, persistent autonomic dysregulation may disrupt homeostatic signalling, generating a sustained state of physiological disequilibrium that perpetuates psychological symptoms [29]. This reciprocal coupling likely underlies the edges observed between psychological factors and physical symptom nodes in our network, creating a self-sustaining symptom network. Interventions targeting autonomic regulation—such as heart rate variability (HRV) biofeedback and transcutaneous vagus nerve stimulation—may thus offer promising therapeutic avenues for affected individuals. Preliminary findings from small-scale pilot studies in LC suggest that these approaches are feasible and associated with improvements in post-viral symptoms, including fatigue, cognitive function, and mental health [30, 31]. To investigate this notion, future studies should incorporate objective markers of autonomic regulation and apply longitudinal designs to map cross-cluster symptom trajectories over time.

Lack of self-efficacy showed moderate associations with COVID-19-related post-traumatic stress and anxiety—a symptom with pronounced centrality in the present network. Prior studies linked higher self-efficacy to lower levels of trait anxiety [32], and meta-analytic findings highlight its protective role in the development of post-traumatic stress symptoms [33]. In the context of LC, affected individuals report lower self-efficacy compared to healthy controls [34], and higher self-efficacy has been related to reduced overall symptom burden [35]. Although self-efficacy did not emerge as a central node in our network, its connection to highly central symptoms suggest it may shape symptom dynamics indirectly. Enhancing self-efficacy could therefore represent a valuable adjunct to interventions targeting core psychological factors in LC.

The present network also revealed positive associations between female gender and gastrointestinal symptoms, and between advanced age and cardiologic and respiratory symptoms, pointing to potential demographic risk patterns in LC. Female gender is a known risk factor for LC [S2], yet evidence on gender-specific symptom profiles remains inconclusive. Past studies report no gender difference in gastrointestinal symptoms [36], whereas others indicate a higher prevalence of gastrointestinal manifestations among females [37]. Similarly, advanced age has been linked to increased risk of LC [S2]. Although evidence for age-related differences in cardiologic and respiratory symptoms remains limited, prior literature support the present association with advanced age [38]. However, interpretations must remain cautious given the underrepresentation of older adults in the current sample. Future studies should employ longitudinal cohort designs with adequate representation across age and gender, enabling stratified and interaction analyses to identify group-specific symptom constellations.

### Limitations

This study has several limitations. First, the present data are cross-sectional, which limits the ability to draw causal conclusions. Specifically, we were unable to confirm past SARS-CoV-2 infection through molecular, or antigen testing. As a result, we cannot rule out alternative explanations, such as other post-viral conditions. A further limitation concerns the broad inclusion criteria, which encompassed individuals with single persistent symptoms alongside those with complex, multi-system presentations; subgroups that presumably differ in their overall disease burden. Additionally, the online self-report design may have introduced selection bias by underrepresenting severely impaired individuals unable to participate. Consequently, the observed network structure may not fully capture the dynamics specific to severe LC populations, limiting generalizability. Future studies that stratify analyses by symptom severity and functional impairment are therefore warranted. Third, we lacked data on pre-existing physical and mental health conditions. Prior research shows that conditions such as anxiety, depression, type 2 diabetes, and cardiovascular disease increase the risk of developing LC [S2]. Without this information, we cannot determine whether the observed symptom patterns reflect consequences of SARS-CoV-2 infection or pre-existing vulnerabilities. Finally, although the network identified key treatment targets, there is limited empirical evidence that interventions targeting these central or bridging nodes lead to reduced symptom burden in LC. Interventional studies adapted to meet the specific needs of individuals living with LC, are needed to evaluate whether modifying these nodes can disrupt maladaptive symptom interactions and improve clinical outcomes.

## Conclusion

This psychological network analysis revealed an interconnected symptom structure in LC, with cohesive clusters of physical and psychological symptoms embedded within an integrated system. Depression emerged as the most central node, underscoring its pivotal role in the overall symptom network. Additionally, depression, alongside cardiovascular, respiratory, and neurological symptoms, served as a bridge between physical and psychological domains, potentially indicating shared underlying pathophysiological mechanisms. Although self-efficacy displayed only moderate connectivity, its links to highly central psychological symptoms suggest an indirect influence on symptom dynamics. Together, these findings emphasize the importance of psychological factors—particularly depression—in LC and advocate for their inclusion in future treatment approaches. Longitudinal and interventional studies are warranted to examine symptom trajectories and assess the effectiveness of mechanism-based interventions.

## Supplementary Material

ckag038_Supplementary_Data

## Data Availability

The data and analytic code underlying this article are available in Open Sience Framework (osf.io), at https://dx.doi.org/10.17605/OSF.IO/CBFHQ [S14].
