# Supplementary material for: Understanding the role of psychological factors in long COVID: a network analysis approach
Source: Eur J Public Health. 2026 Mar 20;36(2):ckag038. doi: 10.1093/eurpub/ckag038 (PMC13017838; doi:10.1093/eurpub/ckag038)
Supplement: ckag038_Supplementary_Data [file ckag038_supplementary_data.docx]

**Supplementary Material**

[Derivation of physical symptom domains: exploratory factor analysis 2](#_Toc221892928)

[Detailed information on the instruments used for assessing the psychological factors 3](#_Toc221892929)

[Table S1. 4](#_Toc221892930)

[Figure S1. 5](#_Toc221892931)

[Figure S2. 6](#_Toc221892932)

[Table S2. 7](#_Toc221892933)

[Figure S3. 8](#_Toc221892934)

[Figure S4. 9](#_Toc221892935)

[Supplementary references of the methods section 10](#_Toc221892936)

# **Derivation of physical symptom domains: exploratory factor analysis**

We conducted an exploratory factor analysis using principal component extraction and Varimax rotation with Kaiser normalization to group individual physical symptoms obtained with the CIS questionnaire into domains. Two symptoms (*fever* and *loss of appetite*) were excluded prior to factor analysis as they were considered non-specific general symptoms that can arise from conditions affecting multiple organ systems. The Kaiser-Meyer-Olkin measure was .801, and Bartlett’s Test of Sphericity was significant (*p* < .001), indicating sampling adequacy. The analysis revealed five symptom domains: (1) neurological symptoms: difficulties concentrating, memory loss/confusion, vertigo, muscle ache, fatigue, headache; (2) cardiological and respiratory symptoms: chest pain, shortness of breath, cough, palpitations, sore throat; (3) gastrointestinal symptoms: nausea/vomiting, stomach-ache, diarrhoea; (4) sensory symptoms: loss of taste, loss of smell; and (5) psychological symptoms: anxiety/worries, low mood, sleep disturbances. The factor loading matrix is provided in **Table S1**.

# **Detailed information on the instruments used for assessing the psychological factors**

*Psychological distress* during the past week was measured using the German-validated short version of the Brief Symptom Inventory (BSI-18; [S3]). The instrument comprises 18 items covering three subscales, somatization, depression, and anxiety. Items are rated on a five-point Likert scale. Due to thematic overlap between the *somatization* subscale and post-viral physical symptoms assessed, only the *depression* and *anxiety* subscale scores were included as nodes in the psychological network model.

*COVID-19 related traumatic stress* was assessed over the past week using the German adaptation of the Impact of Event Scale for COVID-19 (IES-COVID-19; based on [S4]; validated by [S5]). The IES is a well-established self-report scale assessing subjective traumatic stress related to a specific life event [S5]. The IES-COVID-19 adapts this instrument to specifically reference the COVID-19 pandemic as the stressor, capturing the severity of pandemic-related traumatic stress. The 21 items were rated on a four-point scale. A total score was calculated by summing all items, with higher scores indicating greater post-traumatic stress related to adverse experiences due to COVID-19 and the pandemic. This total score was included as a node in the network.

*Chronic stress* over the past month was assessed using the validated German version of the Perceived Stress Scale (PSS; [S6]). The PSS includes 10 items across two subscales, helplessness and self-efficacy. Responses were rated on a five-point Likert scale. Self-efficacy items were reverse-coded (subsequently referred to as *lack of self-efficacy*); thus, higher scores indicating greater chronic stress.

**Table S1.** Results from an exploratory factor analysis of post-viral symptom items based on an adapted translation of the Coronavirus Infection Survey Questionnaire Version 8.2.

| **Item** | **Explained Variance (%)** | **Factor loading** | | | | |
| --- | --- | --- | --- | --- | --- | --- |
|  |  | 1 | 2 | 3 | 4 | 5 |
| **Factor 1: neurologic symptoms** | 14.9 |  |  |  |  |  |
| Difficulties concentrating |  | **.804** |  |  |  |  |
| Memory loss/confusion |  | **.717** |  |  |  |  |
| Vertigo |  | **.632** |  |  |  |  |
| Muscle-ache |  | **.572** |  |  |  |  |
| Fatigue |  | **.568** |  |  |  |  |
| Headache |  | **.542** |  |  |  |  |
| **Factor 2: cardiologic/respiratory symptoms** | 12.9 |  |  |  |  |  |
| Chest-ache |  |  | **.745** |  |  |  |
| Shortness of breath |  |  | **.638** |  |  |  |
| Cough |  |  | **.623** |  |  |  |
| Palpitations |  |  | **.602** |  |  |  |
| Sore throat |  |  | **.489** |  |  |  |
| **Factor 3: gastrointestinal symptoms** | 9.9 |  |  |  |  |  |
| Nausea/vomiting |  |  |  | **.747** |  |  |
| Stomach-ache |  |  |  | **.722** |  |  |
| Diarrhea |  |  |  | **.659** |  |  |
| **Factor 4: sensory symptoms** | 9.9 |  |  |  |  |  |
| Loss of taste |  |  |  |  | **.937** |  |
| Loss of smell |  |  |  |  | **.931** |  |
| **Factor 5: psychological symptoms** | 9.3 |  |  |  |  |  |
| Anxiety/worries |  |  |  |  |  | **.821** |
| Bad mood |  |  |  |  |  | **.782** |
| Difficulties sleeping |  |  |  |  |  | **.626** |

*Note. N* = 283. The extraction method was principal component analysis with an orthogonal (Varimax with Kaiser Normalization) rotation.

**Figure S1.** Proportion of the 21 individual post-viral symptoms in individuals with long COVID (LC) based on an adapted translation of the Coronavirus Infection Survey Questionnaire Version 8.2.

**
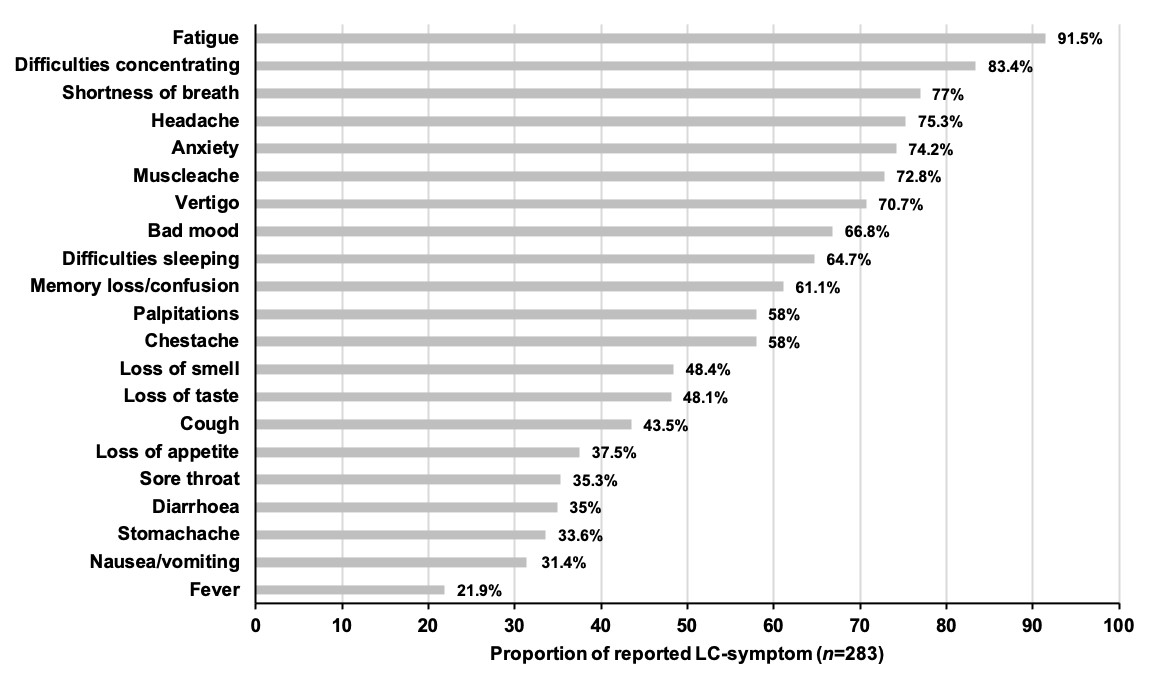
**

**Figure S2.** Bivariate correlations of nodes included in the network analysis

**
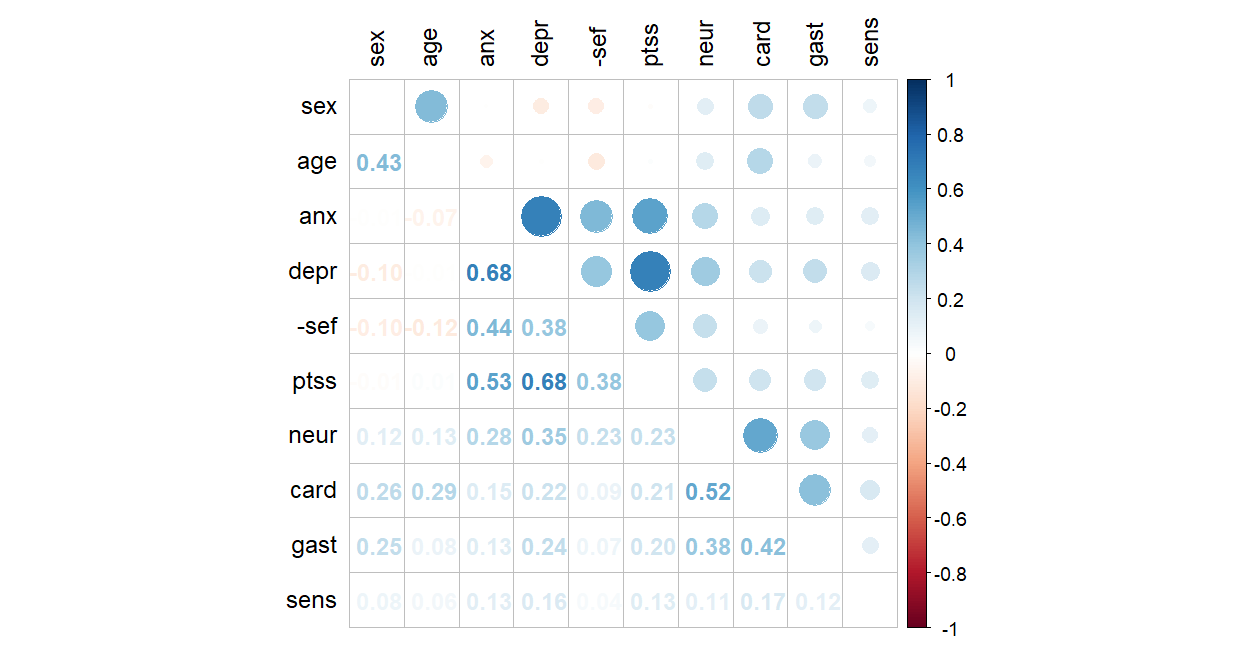
**

**Table S2.** Standardized centrality indices including expected influence and bridge expected influence (z-scores)

| **Node** | **Expected Influence** | **Bridge Expected Influence** |
| --- | --- | --- |
| sex | -0.987 | 0.382 |
| age | -0.754 | -0.121 |
| anx | 0.691 | -1.107 |
| depr | 1.723 | 0.944 |
| sef | -0.561 | -1.102 |
| ptss | 0.449 | -1.024 |
| neur | 0.445 | 1.125 |
| card | 0.872 | 1.351 |
| gast | -0.317 | 0.573 |
| sens | -1.561 | -1.021 |

**Figure S3.** Case-dropping bootstrap showing the stability of the expected influence central index

**
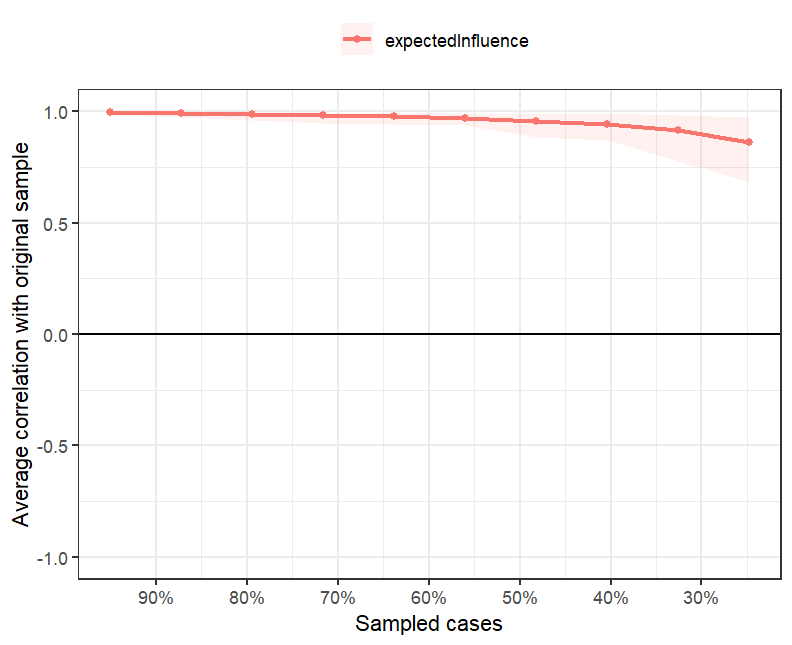
**

**Figure S4.** 95% confidence intervals of edge weights obtained from non-parametric bootstrapping


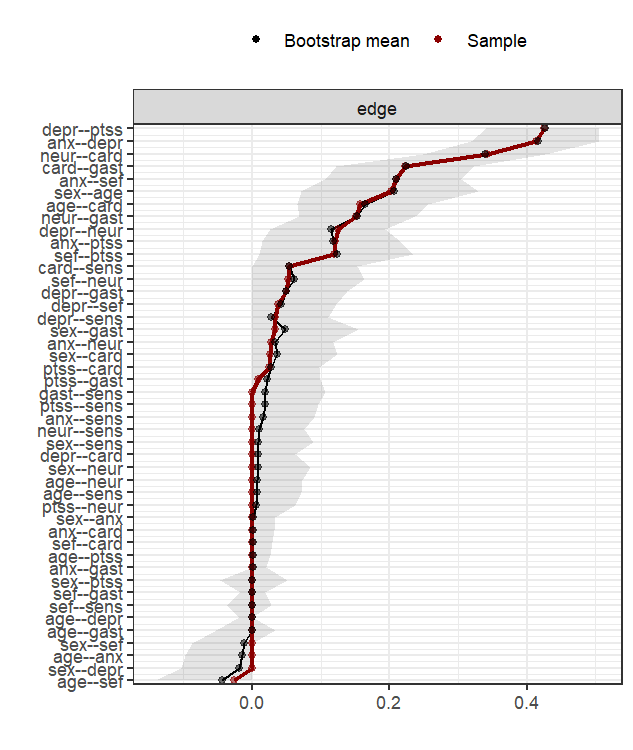


# **Supplementary references of the methods section**

S1. Oehlke S-M et al. Chronic stress and post-traumatic stress in long COVID: a comparative survey study. *Current Psychology*

2025 ***44***:7748–7759. https://doi.org/10.1007/s12144-025-07583-8

S2. Tsampasian V, Elghazaly H, Chattopadhyay R et al. Risk Factors Associated With Post−COVID-19 Condition. JAMA Intern Med 2023;183:566. https://doi.org/10.1001/jamainternmed.2023.0750

S3. Spitzer C et al. Die Kurzform des Brief Symptom Inventory (BSI -18): erste Befunde zu den psychometrischen

Kennwerten der deutschen Version. *Fortschritte der Neurol. Psychiatr* 2011;**79**:517-23. https://doi.org/10.1055/s-0031-1281602

S3. Maercker A, Schützwohl M. Erfassung von psychischen Belastungssfolgen: Die Impact of Event Skala-revidierte Version. *Diagnostica* 1998;**44**:130-41.

S4. Vanaken L et al. Validation of the Impact of Event Scale With Modifications for COVID-19 (IES-COVID19). *Front Psychiatry* 2020;**11**. https://doi.org/10.3389/fpsyt.2020.00738

S5. Klein EM et al. The German version of the Perceived Stress Scale – psychometric characteristics in a representative German community sample. *BMC Psychiatry* 2016;**16**:159. https://doi.org/10.1186/s12888-016-0875-9

S6. Office for National Statistics. COVID-19 Infection Survey CRF5 Version 8.2. https://www.ndm.ox.ac.uk/covid-19/covid-19-infection-survey/case-record-forms (29 September 2025, date last accessed)

S7. Buuren S van, Groothuis-Oudshoorn K. mice : Multivariate Imputation by Chained Equations in R. *J Stat Softw* 2011;**45**. https://doi.org/10.18637/jss.v045.i03

S8. Jones PJ. Networktools: Assorted tools for identifying important nodes in networks. R package version 1.5.0. Published online 2017. https://cran.r-project.org/package=networktools

S9. Epskamp S et al. qgraph : Network Visualizations of Relationships in Psychometric Data. *J Stat Softw* 2012;**48**. https://doi.org/10.18637/jss.v048.i04

S10. Epskamp S, Borsboom D, Fried EI. Estimating psychological networks and their accuracy: A tutorial paper. *Behav Res Methods* 2018;**50**:195-212. https://doi.org/10.3758/s13428-017-0862-1

S11. Friedman J, Hastie T, Tibshirani R. Sparse inverse covariance estimation with the graphical lasso. *Biostatistics* 2008;**9**:432-41. https://doi.org/10.1093/biostatistics/kxm045

S12. Chen J, Chen Z. Extended Bayesian information criteria for model selection with large model spaces. *Biometrika* 2008;**95**:759-71. https://doi.org/10.1093/biomet/asn034

S13. Robinaugh DJ, Millner AJ, McNally RJ. Identifying highly influential nodes in the complicated grief network. *J Abnorm Psychol* 2016;**125**:747-57. https://doi.org/10.1037/abn0000181

S14. [dataset] Oehlke S-M et al. 2025, Understanding the role of psychological factors in long COVID: a network analysis

approach, Open Science Framework (osf.io), https://dx.doi.org/10.17605/OSF.IO/CBFHQ
